# Video Calls for Older Adults: A Narrative Review of Experiments Involving Older Adults in Elderly Care Institutions

**DOI:** 10.3389/fpubh.2021.751150

**Published:** 2022-01-14

**Authors:** Bérangère Naudé, Anne-Sophie Rigaud, Maribel Pino

**Affiliations:** ^1^Université de Paris, Maladie d'Alzheimer, Paris, France; ^2^Services de gériatrie 1 & 2, AP-HP, Hôpital Broca, Paris, France

**Keywords:** older adults, video calls, elderly care institutions, social isolation, health technology assessment (HTA)

## Abstract

Social isolation in geriatric institutions is a real threat to older adults' (OAs) well-being. Visits from family members, when they are not impacted by geographical distance or illness, sometimes fail to provide sufficient opportunities for social connectedness and interaction to prevent and/or combat OAs' loneliness and social isolation. Information and Communication Technologies (ICTs) offer promising solutions to this problem. Video calls provide a quick and convenient way for remote communication between OAs and their families, and a complement to face-to-face visits in geriatric settings. Over the last months, during the several confinements imposed to stop the transmission of COVID-19 over the world, several care homes and long-care facilities have equipped themselves with laptops, tablets and video call applications to help OAs remain in contact with their relatives. However, numerous technical and human-related factors may hinder the use of video calls in these settings. The complexity of technological devices, as well as OAs limited digital skills, low confidence and experience in the use of technology are some examples. Furthermore, the specific context of use and the required implication of multiple actors (care professionals, family members) should also be considered when examining the use and implementation of video calls in geriatric institutions. We conducted a narrative review of literature describing the use of video calls in geriatric institutions between 2000 and 2021, especially because of the little information related to OAs' use of video calls in geriatric settings. One thousand one hundred ninety-seven references were screened and 15 studies focusing on the usability, acceptability and effectiveness of video calls were included. A qualitative, deductive thematic analysis inspired by a Health Technology Assessment (HTA) multidimensional model was used to identify barriers, enablers and solutions to video calls implementation in geriatric institutions. The results from the HTA-based analysis provide encouraging evidence for the feasibility of video call use in geriatric settings, and its efficacy on reducing social isolation among residents. However, numerous technical, human-related, ethical and organizational barriers persist and should be addressed in future works. The present analysis has also allowed the identification of potential solutions to overcome these barriers, which are discussed in this publication.

## Introduction

Social isolation and loneliness represent a serious issue among older adults living in geriatric institutions. Current literature shows that the prevalence of loneliness among nursing home residents is estimated between 50 and 55% (1–3). Indeed, the difficulties to establish new relationships due to health conditions and/or the need for functional assistance (4), or the decline of family visits over time (5), contribute to loneliness and social isolation in this population, at the expense of their well-being (6), quality of life (7) and cognitive functioning (8). With the COVID-19 pandemic and the resulting lockdowns and confinements, elderly care institutions in many parts of the world were instructed to stop all social activities that might put residents at risk (9). As a result, this population, already affected by problems of social isolation, has seen the number of visits decrease drastically (10), increasing their feeling of loneliness and abandonment (11, 12). In an attempt to help OAs maintain richer social interactions at distance than those that may be possible with traditional telephone calls, many geriatric institutions took up or renewed their interest for video calls.

Video calls are a remote communication service offered by several software programs such as Skype (13), Zoom (14) or WhatsApp (15), used to speak with other persons and see them simultaneously on video. Several technologies support these video communications services such as videophones, computers, tablets, smartphones and more recently, mobile telepresence robots (16). The diversity of technological supports, now available for video calls, has introduced new types of user interfaces (e.g., touch, graphical, vocal) (17). In this way, physical pushbuttons and handsets have been progressively replaced by computer mouse, touchpads or touch screens of various sizes. Those technological advances have changed the way users interact with the devices, requiring cognitive and physical capacities which can decrease with age especially if older adults have somatic sensorial or cognitive disorders (18). The rapid development and accessibility of these technologies over the past years has favored the use of video calls (19, 20). More recently, the COVID-19 pandemic contributed to popularize them by highlighting their value as social connector (21), especially as OAs living in geriatric institution lose their traditional social ties. However, a wide availability of these Information and Communication Technologies (ICTs) does not lead to an adoption in elderly care institutions (22–27). Evidence suggests that many technological, individual and contextual factors can influence the use and adoption of ICTs by OAs (10, 28).

Lee and Coughlin (29) identified 10 factors that determine OAs' use and adoption of ICTs products and services, among them: the perceived usefulness and potential benefits of the technology (value), the ease of learning and use (usability), its perceived costs (affordability), its availability (accessibility), the possibility of receiving help if needed (technical support), support received from family, peers and community (social support), the perception of emotional benefits (emotion), the perception of how a technology makes them look to others (independence), OAs' level of experience and confidence in using the technology. According to these authors, these factors are interrelated and have a collective influence on technology acceptance, use and adoption. In the specific case of video call systems used in institutional care contexts, another aspect that must be considered is the required involvement of several actors for the use of the technology, the resident (OA), the family member(s), and the care worker who usually helps during the video call. The study of the implementation and adoption of video call services in the context of geriatric institutions must accordingly consider the perspectives of the multiplicity of actors.

Understanding the barriers and enablers to using video calls in a specific context, such as geriatric care institutions, requires conducting a multidimensional analysis considering, for instance, human, organizational, technological and ethical aspects. However, a few articles in this field have tackled those aspects using a comprehensive approach (30, 31). A recent literature review used a multidimensional analysis method to examine factors for success or failure of mobile telepresence robots' implementation, including video calls functionalities, with OAs at home or in institutions (31). However, the review focused more on the robotic technology itself, than on the implementation of related services. Schuster and Hunter (30), in a scoping review, offered a global analysis of the implementation of video calls in geriatric institutions. Their objective was to describe the use of video calls with institutionalized cognitively intact OAs. Results from the analysis of five studies suggested that video call systems were useful to improve connectedness between OAs and families. However, the perspectives of each actor and their particular barriers were only very briefly described. The authors suggested that further works should explore contextual factors that would help to better understand the feasibility for video communication from the institution perspective (e.g., training needs, organizational aspects), especially as video calling technologies could be valuable tools to fight loneliness, provided that they are implemented strategically (19).

In this sense, the multidimensional analysis models used in the field of Health Technology Assessment (HTA) could provide an analysis grid that questions the clinical, human, technological, medico-economic, ethical and legal aspects of an intervention. This analytical framework makes it possible to assess the global value of a health technology (i.e., its properties, and the effects and repercussions of its implementation) (32). It could be therefore interesting and relevant to study the use of video calls in institutions for OAs using these multidimensional HTA analysis models.

The objective of this review was first to identify barriers, enablers, as well as solutions for the implementation of video calls in elderly care institutions, using a multidimensional HTA approach for the analysis of experimental results presented in publications in the literature, and second to explore the benefits of this service on the maintenance of OAs' social interactions.

## Materials and Methods

### Data Sources and Search Strategy

The aim of this literature review was to analyze published studies describing the experimentation of video call interventions in elderly care institutions. Based on the PICo (population, interest, context) method (33), we developed the following research questions: “*What are the barriers, enablers and benefits for the use of video calls by OAs in elderly care institutions and* w*hat solutions could be considered to overcome those barriers and take advantage of those enablers?*.” The keywords for conducting the search were grouped into three categories: *elderly, nursing homes* and *video calls*. A systematic search was first conducted consulting the following databases: PsycINFO, PubMed/Medline, Web of Science and Scopus. The search was carried out between March and June 2021. We reviewed studies published between January 2000 and June 2021. As with Schuster and Hunter (30), we allowed for a very broad span of time for the inclusion of publications (2000–2021), especially because of the little information related to OAs' use of video calls in geriatric settings. We then searched for studies conducted during the COVID-19 pandemic using keywords grouped into four categories: *elderly, nursing homes, video calls* and *COVID-19*.

The criteria for the inclusion of studies in the review were as follows: (a) experimental studies involving OAs using video call technologies and functionalities, (b) studies describing a video call intervention or activity conducted in a geriatric care institution (e.g., nursing home, assisted living, geriatric service or hospice facility), (c) publications describing an experimental study regardless of the study design (e.g., observational study, case control, randomized study, qualitative study), (d) studies written in English or French. Publications were excluded if: (a) participants gave their opinions only based on photos or videos of video call technologies, without actually using them, (b) studies described experimentations using mobile telepresence robots as a support for video calls.

**Figure 1:**
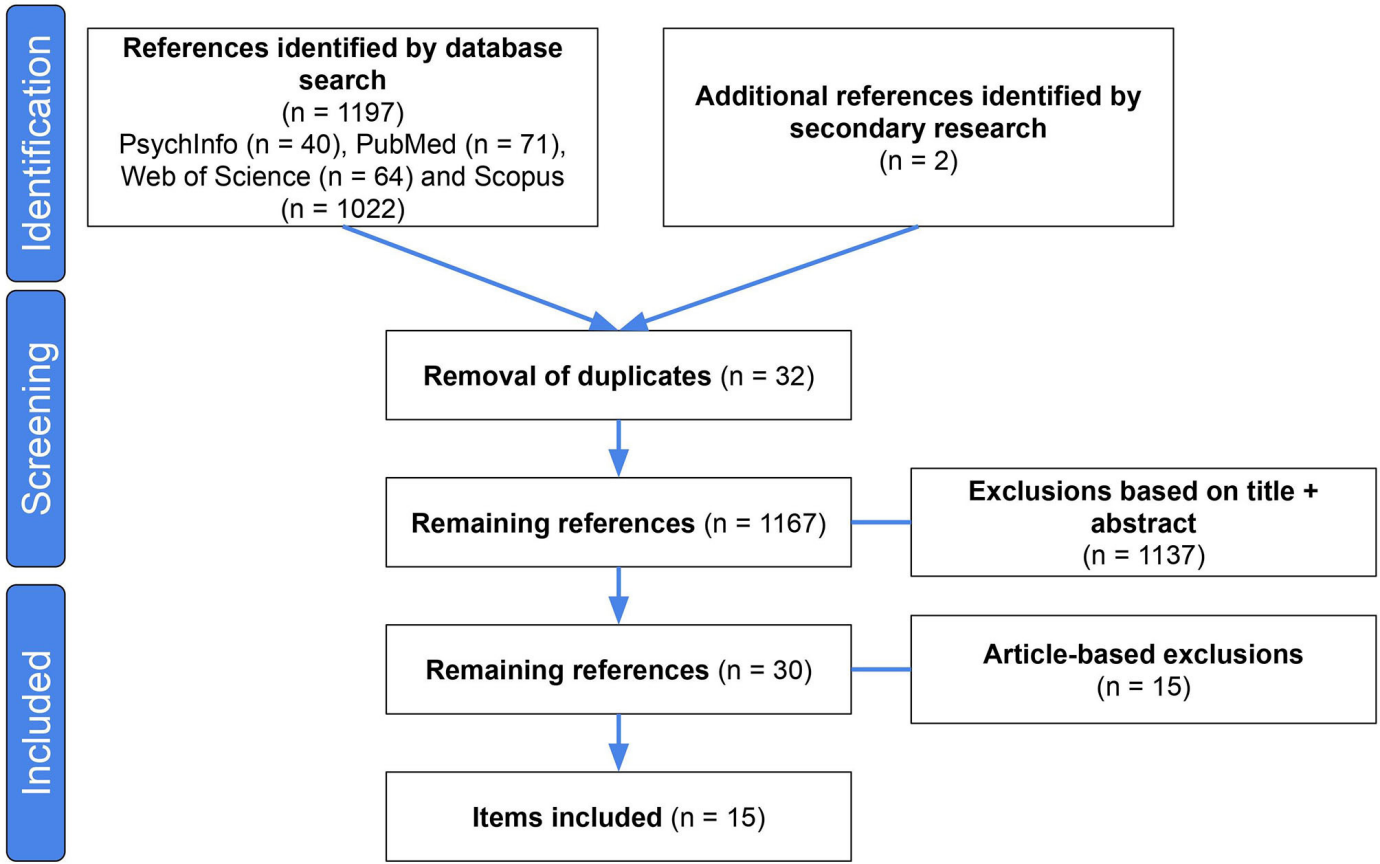
The PRISMA flow chart.

To guide the literature selection process, the Preferred Reporting Items for Systematic Reviews and Meta-Analysis (PRISMA) (34) was used. The study selection was done based on the title, abstract, or full article. Then, a secondary search using the internet and references from other articles was carried out according to the same inclusion criteria. When several publications dealt with the same project, only the publication giving the most detailed information about the work was selected. The flow chart describing the search and study selection strategy is shown in Figure 1.

### The Health Technology Assessment Model

We examined barriers, enablers, as well as solutions to the implementation and use of video calls in geriatric care institutions, as reported in the publications, using the European Health Technology Assessment model (EUnetHTA Core Model®, version 3.0) created by the European Network of HTA (35). Although the main aim of the HTA Core Model is to enable international collaboration in producing HTA information, its ontology can be used in other tasks related to the development, utilization and assessment of health technologies (32). Proper registration of the use of EUnetHTA Core Model®, version 3.0 for this work was made on the HTA Core Model® website (36).

The EUnetHTA core model® version 3.0, is composed of nine domains, each one including several topics, each topic includes as well different issues (i.e., questions that should be considered for the evaluation of a health technology). The model is thus structured into three levels: “Domain” (*level 1*), “Topic” (*level 2*), and “Issue” (*level 3*). The combination of a domain, topic and issue is linked to an assessment element ID, which can be identified using a specific code for standardization purposes (e.g., B0001, B0002…). An example of this combination is presented in Table 1. Main EUnetHTA model domains are: 1. Health and Current Use of the Technology (CUR), 2. Description and Technical Characteristics of Technology (TEC), 3. Safety (SAF), 4. Clinical Effectiveness (EFF), 5. Costs and Economic Evaluation (ECO), 6. Ethical aspects (ETH), 7. Organizational Aspects (ORG), 8. Patient and Social aspects (SOC), and 9. Legal Aspects (LEG). A description of each domain is available in Table 2.

**Table 1:** An excerpt of the Safety (SAF) and Technical Characteristics of Technology (TEC) domains of the EUnetHTA core model.

**Domain**	**Topic**	**Issue**	**Assessment element ID**
*Safety (SAF)*	Patient safety	What are the susceptible patient groups that are more likely to be harmed through the use of the technology?	C0005
*Safety (SAF)*	Safety risk management	How can one reduce safety risks for patients? (including technology-, user-, and patient-dependent aspects)	C0062
*Description and Technical Characteristics of Technology (TEC)*	Features of the technology	Who administers the technology and the comparators and in what context and level of care are they provided?	B0004

**Table 2:** Domains of assessment of the EUnetHTA core model (EUnetHTA Joint Action 2, 2016).

**Domains**	**Main features**
Health and Current Use of the Technology (CUR)	The condition targeted by the technology, the therapeutic purpose of the intervention, and the current standard treatment to address it.
Description and Technical Characteristics of Technology (TEC)	The technical features of the technology, its level of maturity, the resources (material, infrastructural, etc.), and skills required to use it.
Safety (SAF)	The risk and unwanted effects caused by the technology, and the way to prevent and manage it.
Clinical Effectiveness (EFF)	The effects of the intervention on the ability to reach the clinical objectives set for the intervention, on the condition of the quality of life and the autonomy of the users, as well as on the follow up conduct by the professionals who take part in the intervention.
Costs and Economic Evaluation (ECO)	The costs, the health-related outcomes, and economic efficiency of the technology.
Ethical Analysis (ETH)	Issues related to ethics and values when using health technology.
Organizational Aspects (ORG)	The allocation of resources (material artifacts, skills, knowledge, money, work culture, etc.) required to implement the technology in the organization and the healthcare system.
Patients and Social Aspects (SOC)	The representations conveyed by the intervention at the individual's and collective's levels, for the patients, their entourage, the caregivers, and society as a whole.
Legal Aspects (LEG)	The regulations and laws to be considered in evaluating a technological intervention.

### Data Extraction and Analysis

For each publication included in the review, a systematic data extraction was done to summarize: (a) the intervention objectives, (b) the participants' characteristics, (c) the conditions of the experimentations (technology used, duration), (d) the methodology of the study (study design, inclusion of a control group or not assessment tools used), (e) the barriers and enablers to the implementation of the technology (if described), (f) the benefits of the intervention on social interactions (if described), (g) the solutions to overcome barriers to the implementation of the technology (if described).

Then we conducted a theoretical or deductive thematic analysis of the studies using the EUnetHTA Core Model® as a framework. In this “top down” modality of thematic analysis, data is coded and interpreted according to categories or constructs from the existing literature (37). In our case, EUnetHTA domains, topics and issues were used as a set of pre-defined codes to guide the process of data interrogation and organization. Thus, we first identified in each article (methods, results and discussion sections) segments of data that were relevant or captured an idea linked to key concepts of EUnetHTA domains (*level 1*). We proceeded then to a first coding cycle (i.e., label the segments of data). A subsequent exploration of the data coded (sentences or set of statements) was made to get a more analytical identification and defined at the topic level (*level 2*) or at the issue level (*level 3*); corresponding coding was then made using the HTA nomenclature. A semantic approach was used to identify themes and codes using the explicit or surface meaning of the data and not the underlying assumptions or ideas (38). A thematic analysis using EUnetHTA framework for a literature review has been described in another study (31). The 14 selected articles were all coded using this methodology and the software package MAXQDA.

For instance, in the following excerpt from one of the studies selected: “*Residents and family/friends received training on how to use videoconferencing hardware in person and via written materials, respectively*” [(24), p. 320]. Siniscarco et al. described the intervention protocol for using the video calls system, especially the training and information given to the OAs and their family members. Using the EUnetHTA-based coding system, the domain “Description and Technical Characteristics of Technology (TEC)” (level 1) was identified and assigned, then the topic “Training and information needed to use the technology” (level 2) was identified and coded, and finally the issue “What kind of training resources and information should be provided to the patient who uses the technology, or for his family?”(level 3) was also identified and coded. To conclude, the assessment element ID for the corresponding combination Domain, Topic and Issue was added (B0014).

**Figure 2:**
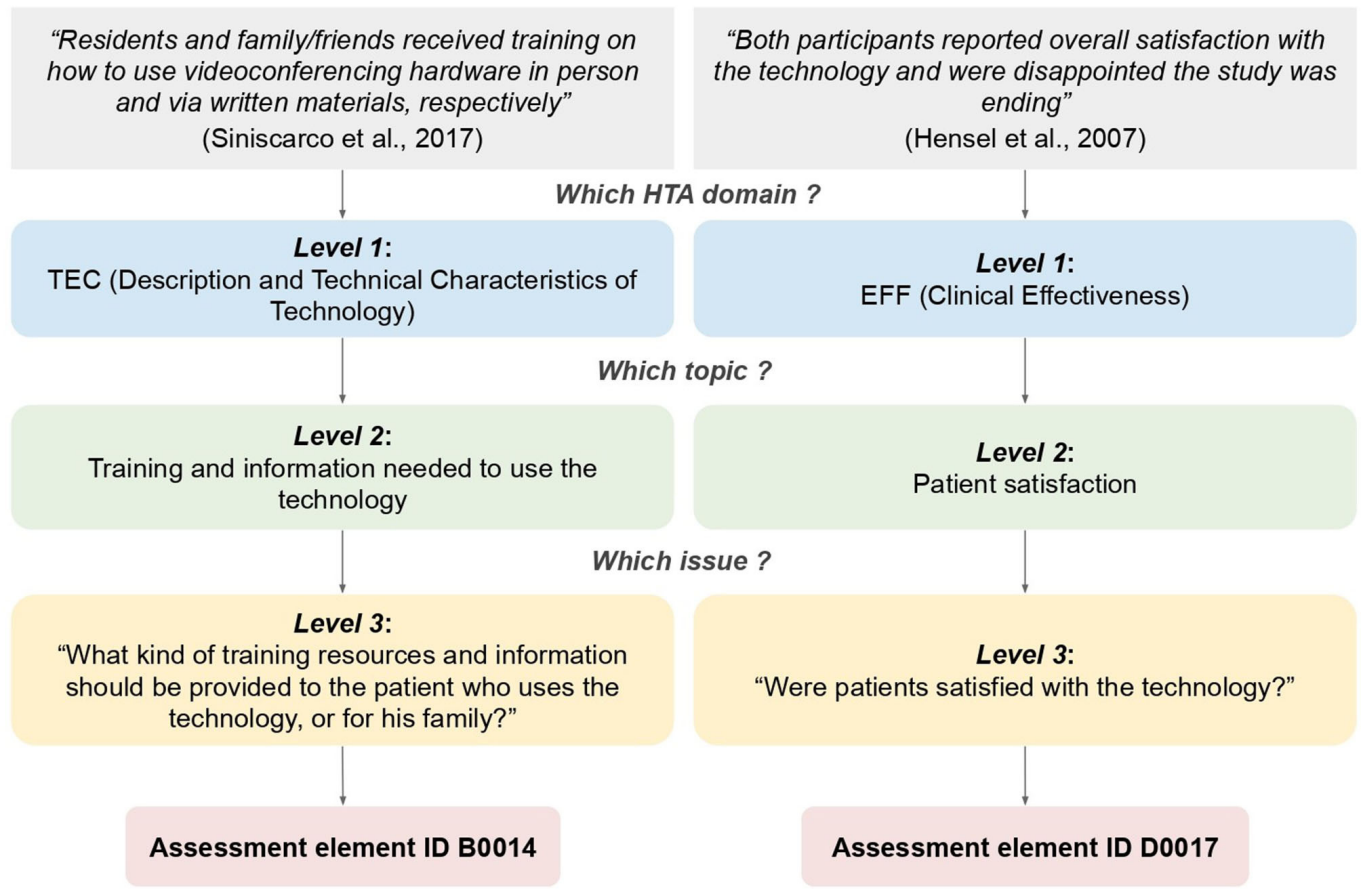
Examples of data coding for the inductive thematic analysis using the EUnetHTA framework.

Following the same methodology, in the following excerpt from another one of the studies selected: “*Both participants reported overall satisfaction with the technology and were disappointed the study was ending*” [(39), p. 124], Hensel et al. claimed that participants were satisfied with the video calling technology. Coding proceeded as follows: EUnetHTA-based domain “Clinical Effectiveness (EFF)” (*level 1*) was identified and assigned, then the topic “Patient satisfaction” (*level 2*) and the issue “Were patients satisfied with the technology?” (*level 3*) were also identified and coded. Finally, the assessment element ID for the corresponding combination Domain, Topic and Issue was added (D0017). Those examples are presented in Figure 2.

## Results

### General Findings

A total of 15 studies were included in this analysis. The studies were published between November 2002 and December 2020. They were conducted in different world regions: North-America (United-States of America) (22, 24, 39, 40), Asia (Taiwan) (23, 27, 41, 42), Europe (25, 26, 43–46), and Oceania (Australia) (47). Regarding the type of institution in which the video calls intervention was conducted, different types of geriatric care institutions were cited: “nursing home” (22–24, 27, 39, 41, 42, 44, 45, 47), “care home” (25, 43, 46), “geriatric hospital” (25, 26, 44) and “assisted living retirement facility” (40). Most video calls interventions used as a support a “touch-screen tablet” (*n* = 5) followed by a “videophone” (*n* = 4), with the remainder using either a “laptop” (*n* = 2), a “smartphone” (*n* = 1), a “tabletop” (*n* =1), or a “TV” (*n* = 2). Three different software programs, such as “Skype” (*n* = 8), “Line” (48) (*n* = 2) and “MSN (Microsoft Social Network, Windows Live Messenger)” (49) (*n* = 2) were used on tablet, laptop, smartphone or TV. The length of experimentation reported in the studies ranged from 1 day to 18 months. A general description of the included articles is presented in Table 3.

**Table 3:** General description of the selected studies.

**Study**	**Country**	**Technology used (application)**	**Time period**	**Type of geriatric care**
Mickus and Luz (2002) (22)	USA	Videophone	6 months	Nursing Home
Sävenstedt et al. (2003) (45)	Sweden	Videophone	3 to 18 months	Nursing Home
Hensel et al. (2007) (39)	USA	Videophone	3 months	Nursing Home
Demiris et al. (2008) (40)	USA	Videophone	3 months	Assisted Living, Retirement Facility
Tsai et al. (2010) (27)	Taiwan	Laptop (Skype, MSN)	3 months	Nursing Home
Tsai and Tsai (2011) (23)	Taiwan	Laptop (Skype, MSN)	12 months	Nursing Home
Siniscarco et al. (2017) (24)	USA	Tabletop (Skype)	2 months	Nursing Home
Zamir et al. (2018) (25)	UK	Tablet (Skype)	15 months	Care Home, Geriatric Hospital
Chiu and Wu (2019) (41)	Taiwan	Tablet (Line or Youtube)	12 weeks	Nursing Home
Moyle et al. (2019) (47)	Australia	Tablet (Skype)	1 day	Nursing Home
Niebler et al. (2019) (26)	Germany	Tablet (Skype)	NP	Geriatric Hospital
Tsai et al. (2020) (42)	Taiwan	Smartphone (Line)	6 months	Nursing Home
Sacco et al. (2020) (44)	France	NP	2 weeks	Nursing Home, Geriatric Hospital
Carcavilla et al. (2020) (43)	Spain	TV (Skype)	6 weeks	Care Home
Zamir et al. (2020) (46)	UK	Tablet and TV (Skype)	8 months	Care Home

*MSN, Microsoft Social Network (Windows Live Messenger); UK, United Kingdom; USA, United States of America; NP, Not Precised*.

Concerning the methodological aspects, the majority of the study designs were “exploratory qualitative studies” (*n* = 5), “randomized trial” (*n* = 3) or “ethnographic and a part of a Collaborative Action Research (CAR)” (*n* = 2), the remaining ones were a “case study” (*n* = 1), a “randomized longitudinal trial” (*n* = 1), an “exploratory mixed-methods study” (*n* = 1), a “cross-sectional study” (*n* = 1), or a “quasi experimental study” (*n* = 1). Regarding the main objective of the assessment described in the studies, more than half addressed the feasibility and acceptance of video calls technologies, notably through the study of their usability and usefulness, the other half studied the clinical impact of video calls on loneliness or depression. Finally, the majority of the studies involved both healthy OAs and OAs with cognitive impairments (*n* = 12). However, the severity of the impairments remained unclear. Only a few publications reported explicitly involving OAs with severe cognitive decline (dementia) (*n* = 3). A summary of the methodological features of the studies is presented in Supplementary Table 1.

### Description of Studies Using HTA Dimensions Including Topics and Issues When Available

In the following section the HTA-based thematic analysis of the studies' findings is presented using the three levels of analysis of the EUnetHTA model, described in Methods. Each segment refers to one of the nine major EUnetHTA domains (CUR, TEC, SAF, EFF, ECO, ETH, SOC and LEG). Then, data that refers to the next two EUnetHTA analysis levels “Topics” and “Issues” are described. The EUnetHTA core model® version 3.0 assessment element ID is provided for each issue described.

**Figure 3:**
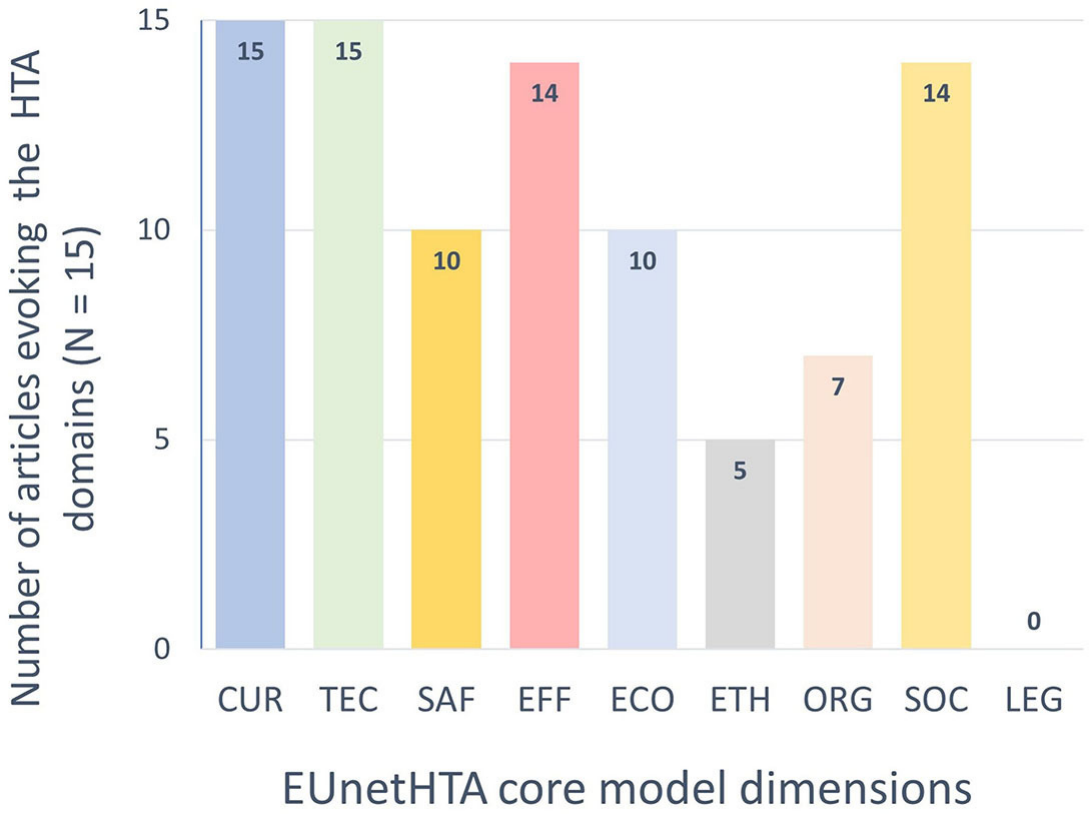
EUnetHTA core model domains occurrences within the 15 selected articles.

A summary of the distribution of HTA domains by articles is shown in Figure 3.

#### Population, Health Problem and Current Use of the Technology (CUR)

##### Target Population

Issue: “What Is the Target Population in This Assessment?” ID (A0007).In all the studies included (100%, *n* = 15), the target population for the interventions using video calls was “OAs living in elderly care facilities.” These institutions were: nursing homes (22–24, 27, 39, 41, 42, 44, 45, 47), care homes (25, 43, 46), geriatric hospitals (25, 26, 44), and assisted living retirement facilities (40). Target populations in the studies were either healthy OAs, suffering from mild cognitive impairment (MCI) (22–25, 27, 39–43, 46, 47), or a major cognitive impairment, including dementia (26, 44–46).

##### Target Condition

Issue: “What Aspects of the Consequences/Burden of Disease are Targeted by the Technology?” ID (A0009).The aim of video calls interventions was to target socialization need of OAs living in institutional settings. OAs living in elderly care institutions frequently experienced loneliness, infrequent social contact with their relatives or friends, a lack of meaningful relationships, and/or difficulties in forming new relationships (24, 41, 43, 45, 46).

##### Current Management of the Condition

Issue: “What are the Other Typical or Common Alternatives to the Current Technology?” ID (A0018). Before the implementation of video calls in elderly care institutions, OAs already had their own habits, like regular in-visits or telephone calls. Several studies refer to existing modes of socialization with relatives that may compete with video calls. Most of the studies mentioned face-to-face visits (22, 25, 26), followed by telephone calls (22, 47), and finally letters (22).

##### Utilization

Issue 1: “For Which Health Conditions and Populations, and For What Purposes Is the Technology Used?” ID (A0001). Video call interventions were offered to OAs living in geriatric care facilities to reconnect with families, facilitate interactions, or maintain social connectedness (23–25, 27, 40–42, 45). The other publications did not mention any precise utilization purpose of video calls (22, 26, 39, 43, 44, 46, 47).

Issue 2: “Is the Technology a New, Innovative Mode of Care, an Add-on to or Modification of a Standard Mode of Care or Replacement of a Standard Mode of Care?” ID (F0001). In two studies, video calls were considered to be an additional means of long-distance communication (26, 47), and one better than the traditional telephone (22). In cases where the family could not visit their loved one, or where such visits were too demanding for them, video calls could be used to replace them (22, 39, 44). Finally, video calls could also be part of a leisure activity offered to residents by staff members (43, 46).

#### Description and Technical Characteristics of Technology (TEC)

##### Features of the Technology

Issue 1: “What is This Technology and the Comparator(s)?” ID (B0001). Studies experimented and assessed video calling technology. Video calls allow face-to-face contact during a call (associating audio and video) and can be used on different technological supports. Among the 15 studies reviewed, six different video calling technologies were used, as well as three different freeware programs. Two of them are instant messaging and video calling applications (e.g., MSN, Line), while the other one provides only video calls services (e.g., Skype). Detailed descriptions of these technologies, how they were used, and in which settings are presented in Supplementary Table 2. One study specified neither the equipment nor the software program used (44), six others did not specify technical issues (23, 27, 41–43, 45), and three did not specify the setting (27, 40, 43), and frequency of use (22, 26).

Issue 2: “Who Administers the Technology and the Comparators and in What Context and Level of Care are They Provided?” ID (B0004). For the use of video calling technology, OAs were either independent (22, 39, 42, 44), partially assisted by facility staff (22–24, 27, 41, 42, 44, 47), or completely dependent on external help (25, 26, 45, 46). In some cases, even if the OA was independent when using the technology, it was usually family members who initiated the calls (22–25, 39, 45).

Issue 3: “What Is the Claimed Benefit of the Technology in Relation to the Comparators?” ID (B0002). After introducing video calling technology and services in the geriatric facilities, residents, families and care staffs reported different benefits. Among them, the most cited was the fact of being able to have richer and more emotional conversations, compared to traditional calls, by the addition of the video dimension (22, 39, 40, 44–46). This visual dimension seemed to provide a real psychosocial support to the resident (26). In this regard, video call interventions could become an integral part of the care process. Video calling was also helpful to overcome “social barriers” and help OAs to reconnect with their families and friends when they had been distant and had no or little contact (41). Another advantage of video calling technologies was the possibility of using them anywhere thanks to their mobility (25, 42).

##### Investments and Tools Required

Issue: “What Material Investments are Needed to Use the Technology?” ID (B0007). Video calling intervention implies some material investments (e.g., hardware, software, Wi-Fi access, supports…). Supplementary Table 2 describes the equipment and services used in the studies for conducting video call interventions. In some studies, basic video calling technology was “disguised” or furnished to improve the user experience and perceived usability by residents. Four studies using the tablet included a support to avoid the need for the resident to hold it [e.g., wheel support (25, 46), traditional support (26, 41)], and three added a traditional handset to reassure the participants about the video calls, but also to help them understand the use of the tool (25, 26, 46). Finally, only one study proposed a sensor pen in addition to the tablet support to facilitate the use of the touch screen (41), and two others had to improve the ergonomics of the tool to make it more accessible (large, bright, raised numbers on the keypad or phone with volume control) (22, 24).

##### Training and Information Needed to Use the Technology

Issue: “*What Kind of Training Resources and Information Should be Provided to the Patient Who Uses the Technology, or For his Family?” ID (B0014)*. In order to correctly use video call technology and services, training or information sessions may be necessary for users. Among the selected studies, six did not specify this information (39, 40, 43–46), and four did not provide information to the OAs, with staff members being designated to use the technology for them (22, 23, 25, 26). Only seven studies devoted time at the beginning of the experiment to, at a minimum, informing OAs about how video calls work on the designated tool. In one study, the researchers preferred one-on-one training where they first showed the resident and family how the tool worked, rehearsed just before the first call, attended the first call, and finally provided a reminder of the instructions on paper (22). A second study preferred a group training (about 10 OAs), with a 1.5-h session every week for 12 weeks. These sessions consisted of general information about the tool and its functionalities, but also practical exercises with reminders at the beginning of each session about the actions seen in the previous session (41). In the remaining studies, the researchers simply showed, informed, or had the resident practice once before the video call intervention took place (24, 26, 27, 42, 47).

#### Safety (SAF)

##### Patient Safety

Issue: “What are the Susceptible Patient Groups That are More Likely to be Harmed Through the Use of the Technology?” ID (C0005). Regarding the safety of OAs who took part in the video call intervention, some staff members expressed concerns about physical risks linked to the use of video call technologies [e.g., fear of hurting residents when moving the “Skype on Wheel” (SoW) tool (a tablet on a wheeled support) through the hallways] (25). In addition, some unwanted and harmful psychological risks were described, for instance, a professional emphasized the anxiety-inducing aspect of the tool when entering the resident's room. Indeed, one resident showed anxiety and confusion when he saw the tool arrive in his room (25).

Also, regarding psychological risks, it was noted that many participants expressed frustration or even confusion with the many connection, audio, or video issues that disrupted calls (22, 24, 25, 40): “*she grew concerned that her family did not want to speak to her*” (25).

Furthermore, the technology itself could be intimidating some OAs, confronting them with their own physical and/or cognitive difficulties and disabilities (24, 25, 41, 47). One resident was afraid of “*looking silly trying to use video calls*” (25). This apprehension of new technologies may also echoed their perceived vulnerability, to something they did not control or understand. An example of this was the fear of having their own identity stolen by hackers (26, 47).

Finally, some residents with dementia expressed fear that video call technology would replace visits from their loved ones (45), who would see it as a way to alleviate their obligation to visit their relative (26). Finally, these same residents did not always seem to understand the concept of video calls. Although they recalled talking to other OAs, they did not recall using a videophone tool (46). This discrepancy could lead to confusion among these already anxious residents (25).

##### Safety Risk Management

Issue: “How Can One Reduce Safety Risks For Patients (Including Technology-, User-, and Patient-dependent Aspects)?” ID (C0062). To reduce the risk of confusion and anxiety about the video call technology, some care staff suggested “disguising” the video call equipment. The goal was to make it more user-friendly (25). As far as technical problems are concerned, no solutions were discussed in the publications. As the problems were mostly due to a bad internet connection, there was no way to resolve them immediately and in real time (38). On the other hand, audio and video problems seem to have decreased or even disappeared with the appearance of tablets, computers and smartphones.

#### Clinical Effectiveness (EFF)

##### Morbidity

Issue: “How Does the Technology Affect Symptoms and Findings (Severity, Frequency) of the Disease or Health Condition?” ID (D0005). The use of video calls in an elderly care facility can have an effect on health outcomes of the target population. Two studies found a decrease in depressive symptoms experienced by most residents (15, 19, 34). Another study found a positive effect on pain, vitality and physiological health of OAs (34).

##### Health-related Quality of Life

Issue: “What Is the Effect of the Technology on Generic Health-related Quality of Life?” ID (D0012). The use of video calls can also affect the health-related quality of life. The main reported benefits of its use were the improvement of the users' well-being (24, 41, 46), a better self-perception (42) and self-esteem (43). Thus, some participants reported feeling younger or feeling in tune with the younger generation. In addition, the use of video calls was also associated with a decrease of loneliness (23, 24, 27, 42), and social isolation (24). Other benefits on the general well-being of the person were also observed, such as an improvement of quality of life (mental component) (41).

Regarding the interactions between the participants and their relatives, video calls seemed to improve family and friend social support (41). The effect of video calls on the quality and quantity of social interactions were not unanimously acknowledged by the authors. Some observed an increase in the number of social interactions (40, 46) and/or in social connectedness (22, 40, 46), especially regarding remote communication in non-verbal participants (22), while other observed no changes, either in terms of quantity (22), or quality (23). Some OAs who benefited from video calls reported a feeling of closeness with the family, such as the feeling of “*still [being] part of the family*” (40).

Beyond the feeling of being integrated, a real feeling of presence was associated with the use of video calls. Indeed, many participants reported that: “*It was like having him [the family member] in the room with me [OA]*” (24); “*The visual aspect helped me to feel like I was visiting when we spoke*” (40). This feeling of presence was stated in four studies (23, 24, 39, 40). Through the improvement of emotional support (15, 16, 19), informational support (24), and appraisal support (Social Support Behaviors scale) (23, 27), video calls finally helped to reassure elderly participants about the health status of their relatives (39).

Finally, video calls in general offered a new activity, a distraction to the residents to combat boredom and to help them “pass the time” (31). Thus, residents seemed to “regain a sense of self and purpose again” (46).

##### Patient Satisfaction

Issue: “ *Were Patients Satisfied With the Technology?” ID (D0017)*. Studies that reported users' feedback (OAs and family members) described a good level of satisfaction of using video calls to communicate with their loved ones (22, 24, 26, 39–41, 44, 45).

In one study, a family member declared that his elderly relative seemed more relaxed and focused on the conversation during video calls than in traditional visits (45). A study reported that OAs looked forward to occupational activity sessions using video calls, since these were integrated into a leisure and game context between nursing homes where “*winning became our home's pride*” (46). Indeed, some authors suggested to embed video calls sessions into a more global social interaction activity such as arranging for family members to have a meal together with residents via videoconferencing (23).

Finally, a few studies reported that some participants rejected video calls, notably because of technical problems previously described in section Description and Technical Characteristics of Technology (22, 24), but also because of a poor acceptance of the equipment itself (26). The situation could then quickly become a source of anxiety for the residents, some showing confusion caused by the perceived complexity of the technology, among other things (25, 26). Finally, one participant did not see any value in video calls because of her vision impairment (22).

#### Organizational Aspects (ORG)

##### Health Delivery Process

Issue 1: “How Does the Technology Affect the Current Work Processes?” ID (G0001). The implementation of video calls in geriatric institutions may require the involvement of facility staff members, which impacts their work and the care process. In eight studies, staff members assisted (22, 42, 44), or organized and implemented video call interventions (23, 25, 26, 45, 46) in addition to providing care to OAs. The use of video call technology added an additional task to an already busy schedule, increasing their workload (25, 26, 46). In one study, staff members declared as well that their priority was to ensure physical care: “*families need us [staff members] to focus on the care*” (46). Depending on the conditions imposed by experimentation, video calls could be made once a day (44, 45), once a week (23, 42), or once a month (25, 46).

Issue 2: “What Kind of Process Ensures Proper Education and Training of Staff?” (G0003). To ensure proper assistance to OAs with the use of video conferencing technology, researchers trained staff members (23, 25), held an event to introduce the technology (26), or simply demonstrated how it works to staff members (22).

##### Structure of Health Care System

Issue: “What Are the Processes Ensuring Access to the New Technology For Patients/Participants?” ID (G0101). In cases where facility staffs were directly involved in the use of video calls, different processes were implemented. Some facilities practiced up-front appointment setting between the family and the resident (23, 42, 44). In one case, facility staffs helped relatives know which was the best time to call the OA (awake and alert), and informed the relatives of the OA's health status: “*part of the time I [family member] get an update from her [staff member] on what is going on with regard to my husband*” (45). Staff members were also especially helpful in reassuring participants about the video calling technology (44).

Sometimes, the use of video calls was considered as another social activity and therefore was presented on regular communication supports of the institution (newsletter to the families) (25). In the case of the use of video calls for a game competition activity between the geriatric residences, the staff members were responsible for organizing the sessions and ensuring that the equipment worked properly (46).

##### Management

Issue: “What Management Problems and Opportunities are Attached to the Technology?” ID (G0008). The integration of a new technology into an elderly care institution often raised management issues. When facility staff was asked to implement video calls, understaffing and an already heavy workload were recurring organizational issues (25, 26, 46). Staff turnover and changing roles could also lead to loss of important information for the use of video calls, but also to a loss of skills (25, 46).

##### Culture

Issue: “How Is the Technology Accepted?” ID (G0010). The success of the integration of a new technology, activity, or intervention depends partly on its acceptance by facility staffs. In two studies reviewed, professionals showed no interest in ICT technologies and did not understand their usefulness in an OAs facility (26, 45). This lack of interest sometimes turned into an aversion to the technology, and despite training, professionals found it difficult to appropriate it, representing supplementary workload. A few professionals felt intimidated and considered video calls as a burden (25, 26). Some also doubted about their ability to learn how to use a new technology, which directly impacted their commitment (25).

However, when these professionals were involved as real actors in the video calls activities, and not only as assistants, the appropriation of the technology was better (46). In one study, organizing, participating, and observing the firsthand benefits of the technology increased professionals' commitment and desire to continue using it; especially since the intervention provided an opportunity for staff to “*link up [with other residences] and become more connected with each other to provide a more 'close knit' unit”* (46).

#### Cost and Economic Evaluation (ECO)

##### Resource Utilization

Issue: “How Does the Technology Modify the Need For Other Technologies and Use of Resources?” ID (D0023). The introduction of video calling technologies in an elderly care institution needs, at the very least, an investment in the basic video calling equipment (hardware, software). This kind of intervention may create other needs in terms of technology (Wi-Fi coverage in all rooms) but also resources (provision of user guides).

In this sense, beyond the help provided by staff members and researchers, most OAs expressed a need for additional and regular assistance in the use of video conferencing technologies (23, 42, 44, 45, 47), or individual training sessions: “*I would definitely need someone to help”* (39). In addition, the presence of technical problems almost always required the intervention of researchers (22, 24, 46). That is why Moyle et al. suggested to employ skilled staff to assist OAs with videoconferencing (47). However, a few staff members also requested additional guidance in the training provided (25). Finally, problems of accessibility of the technology required the use of additional tools, or ergonomic adaptations (e.g., sensor pens, support, volume control, larger screen) (22, 24–26, 41). Another solution could be to use a telepresence robot allowing the resident and their family to connect *via* a free-standing, wheel-based, videoconferencing system (47), or take advantage of the nurses station for this purpose, where tablets and help could be available for residents if needed (24).

#### Ethical Aspects (ETH)

##### Benefit-harm Balance

Issue: “What are the Benefits and Harms of the Technology For Relatives, Other Patients, Organizations, Commercial Entities, Society, Etc.?” ID (F0011). When implementing a new mode of remote communication involving the use of a technology, it is important to study the balance between the benefits and harms that it generates. First, there is the risk of reducing, or even completely replacing, traditional visits by relatives in the institution (27). Some families saw video calls as a way to reduce their guilt toward their institutionalized relatives or to reduce their sense of obligation to visit him or her, by substituting the visits with video calls (26).

However, no studies reported a decrease in the frequency of traditional visits after the introduction of video calls. On the contrary, several times, video calls were shown to increase social interactions between residents and their relatives, especially when the latter was unable to travel due to health reasons or geographical distance (22, 39, 40, 44).

##### Respect for Persons

Issue: “What are the Known and Estimated Benefits and Harms For Patients When Implementing or Not Implementing the Technology?” ID (F0010). The use of video calls raised the issue of privacy. Indeed, the facility staff members were often requested to ensure proper video calls functioning. This constant supervision raised the question of the privacy of exchanges between family members and OAs (26). In addition, the OAs expressed their concern about the loss of control over their image (47), but also about their perceived vulnerability to cyber-attacks (26, 47).

##### Autonomy

Issue 1: “Is the Technology Used for Individuals That are Especially Vulnerable?” ID (F0005). Some studies included OAs living with dementia or other advanced cognitive disorders (26, 44–46). Some of them expressed confusion and anxiety when the video calling technology was introduced in their room, causing agitation. However, these negative reactions seemed to decrease when the OAs recognized their relatives on the technology's screen (25). In some cases, OAs with dementia did not remember the conversations held during video calls (26). However, in another study, OAs with dementia could remember details of the conversation, the interlocutors, but not having used video calling technology to talk to them (46).

##### Justice and Equity

Issue: “Are There Factors That Could Prevent a Group or Person From Gaining Access to the Technology?” ID (H0012). Some factors may prevent some OAs living in geriatric institutions from taking advantage of or even using video calls. In a few studies, staff members decided which OAs were eligible (i.e., considered capable) for video calls intervention, without giving OAs the opportunity to try or to give their opinions on the video calling technology (24, 25, 47). In one study, researchers explained that this categorization could lead to discrimination. Indeed, researchers observed that some OAs with some cognitive or sensory deficits were naturally excluded, considered unable to use or benefit from the intervention (e.g., non-verbal OAs) (25).

#### Patients and Social Aspects (SOC)

##### Social Group Aspects

Issue: “Are There Factors That Could Prevent a Group or Person From Gaining Access to the Technology?” ID (H0012). Many factors limited access to technology for all facility residents, or only for a group of vulnerable OAs. Their self-perception, their abilities, but also the complexity of the tool are limiting factors to video calls use. Some OAs expressed insecurity about the image they projected of themselves through the use of video calls (25, 46), or had a low self-efficacy, simply not feeling capable of using such a technology: “*for me at 90, it is going to be difficult*” (47), “*Too old for VTC [Video telecommunication]*” (26), “*she would look 'silly' trying to use video calls*” (25). Another factor that could prevent the use of video calls was the attitude of OAs toward technologies in general. A negative attitude toward technologies (26), a feeling of discomfort when using technologies (24), a poor digital culture (44), and a low tolerance toward technical problems (22) were barriers to the use of video calls. On the contrary, a good tolerance toward technical problems was often associated with a good level of engagement with video calls (22). Some OAs who were not interested in this technology did not give any particular reason to explain their choice (25, 27, 45).

Some OAs did not dare ask their relatives to participate, thinking that they would be too busy to do so anyway (22, 24). The family, in fact, as an actor in the implementation of video calls, conditioned its use most of the time. Thus, an OA with relatives lacking involvement would generally not participate or would drop out of the video call study. A limited implication of the family (23, 25) could be explained by the lack of technical skills (22, 25–27), the difficulty of access to the necessary tools (25–27), the lack of motivation and interest in the technology (22, 26), their limited availability (22, 25–27), a poor relationship with their relative (26), preconceptions about the institutionalized person's ability to use the device (e.g., thinking that it would be too difficult for the OA to use such a technology) (25, 27), their dependence on facility staff to make calls (45), or their difficulties in making an appointment to call the OA (if they were not in the same time zone for example) (24). A family that was very present and regularly visited the OA also limited the use of video calls (i.e., low usefulness) (22, 27, 42).

##### Patient's Perspectives

Issue 1: “What Expectations and Wishes Do Patients Have With Regard to the Technology and What Do They Expect to Gain From the Technology?” ID (H0100). No study mentioned the expectations and wishes of OAs toward video conferencing technologies. They only evaluated their opinions during or after the interventions.

Issue 2: “How Do Patients Perceive the Technology Under Assessment?” ID (H0006). Several OAs considered video calls as a way of reconnecting with their family and renewing social ties (23, 25, 26, 47). This new experience (26) was evaluated as positive (25, 39, 47), having the potential to improve the quality of conversations with the family: “*it would feel like you were talking to the caller in-person and be more in contact”* (39). However, video conferencing was still considered the second-best option compared to in-person visits (26, 42). However, some OAs perceived the technology used as intimidating (24), complicated (25), and even dangerous in the case of cyber-attacks (26, 47).

#### Legal Aspects (LEG)

Aspects related to rules and regulations were not described in the publications reviewed.

## Discussion

The purpose of this research was to compile and review existing literature on video calls involving OAs in elderly care institutions from January 2000 to June 2021. Our search identified 15 studies, with a wide variety of intervention designs, study settings, and sample characteristics. The objective of this review was first to identify barriers, enablers, as well as solutions to the implementation of video calls in elderly care institutions using a multidimensional perspective, and second to explore the benefits of this service on the maintenance of OAs' social interactions. The EUnetHTA multidimensional framework (35) was used for guiding the analysis of publications. In this section, we discuss main facilitators, barriers and solutions identified for the implementation of video calls in geriatric settings and provide suggestions for future work. Supplementary Table 3 summarizes enablers, barriers and inferred solutions.

### Factors That Facilitate the Use and Adoption of Video Calls Interventions

#### Feasibility

Among the enablers of video calls interventions, it is important to underline that most of the authors showed the feasibility of the implementation of video calls in elderly care institutions. Evidence of this is that, at the end of each study, despite the barriers encountered (technical difficulties, need to be helped by professionals during the video calls), several OAs continued to benefit from video calls with their relatives. This can be explained by the fact that the participants found the benefits more important than the obstacles.

It is interesting to note that videophones did not elicit strong rejection from residents, as their ergonomics were similar to those of traditional landline telephones, which are widely used among this population. Furthermore, using video calls instead of telephone calls was more a change in the means of communication than a change in the habits of communication for the OAs. Video calls interventions were also considered feasible because devices employed were available for a general public and were also low cost.

#### Usefulness

As emphasized by all the studies in this review, video calls are useful since they enable OAs to have more meaningful remote communication (both audio and visual) with their relatives, especially those who live far away. Furthermore, the several COVID-19 confinements particularly highlighted the usefulness of video calls, as reported in two studies (44, 46). Thus, despite some reluctance of institutionalized OAs toward those technologies, they seemed to prefer video calls over traditional telephone calls to communicate with their relatives (44).

#### Motivation

Another strong enabler to video calls use was OAs' interest in video calls service. However, as their environment played a central role, OA's motivation was closely related to family and staff members' motivation itself. Indeed, those two stakeholders played an active role in facilitating the use of video calling technology by OAs. However, family and staff members' implication and interest on the service mostly depended on their capacity of using it, on their tolerance of technical and operating difficulties (14), as well as on their attitudes toward video calls. Most of the time, their positive attitude would reflect on the resident, and thus would encourage video calling technology usage (39). For example, Luijkx et al. (50) showed that OAs easily adopted the enthusiasm of their grandchildren for technology (50). Moreover, the proactivity of family and staff members through stimulations and availability of technical support also participated in video calls use by OAs.

### Video Calls as a Form of Psychosocial Intervention to Support Socialization in OAs

The secondary objective of this work was to explore the impact of video call interventions on social interactions. The results of the present study showed that nine articles out of 15 that were selected identified a positive impact of video calls on the maintenance of social ties, either in terms of improving the quality of interactions and social support between the resident and his/her relatives, or in terms of social isolation and loneliness. These results are in line with those shown by Schuster et al. (30) in a previous work dedicated to video calls for cognitively intact OAs (30). In a review of literature focused on interventions to combat loneliness in OAs living in long-term care, Quan et al. (3) also observed a reduction of loneliness with video conferencing in nursing home residents (3). They categorized video conferencing with family members as a “social facilitation interventions”, according to a classification proposed by Gardiner et al. (51). According to these authors, the primary purpose of this kind of intervention is to facilitate social interaction with peers, or others who may be lonely: “*social facilitation interventions generally presume a degree of reciprocity, and strive to provide mutual benefits to all participants involved”* (51). Such interventions could help OAs to maintain social relationships with family and friends, especially when they are not able to do physical exercise or travel anymore (52). Indeed, residents suffering from a reduction of mobility are at risk of having fewer social relationships and feeling lonely. Quan et al. (3) underlined that the most successful interventions for these OAs were those that did not entail physical activity or mobility (3).

However, although results that evaluate the efficacy of these interventions are promising, there are still few randomized studies on the implementation of video calls in institutions, and most of them involve small samples (30, 53). Thus, no significant evidence was found to support the effectiveness of video calls on reducing loneliness in older adults (53, 54). Future randomized trial with large samples would be necessary to confirm the benefits of video calls interventions involving OAs in geriatric institutions. It is interesting to note that those benefits could be potentiated if video calls sessions with relatives were complemented either by entertaining activities performed individually (41) or in group while videoconferencing with other residents from other institutions (46). Thus, even though “*there is no one-size-fits-all approach to addressing loneliness or social isolation”* [(54), p. 2], future studies should provide concrete guidance for interventions to be more effective with this population.

### Identified Barriers to the Implementation of Video Calls Interventions and Potential Solutions

The implementation of video calls in institutions comes up against several technological, human, organizational and ethical obstacles.

#### Technological Barriers and Possible Solutions

This narrative review has included a broad span of technologies (Supplementary Table 2), from videophones to tablets or smartphones. However, only three different software programs were used (e.g., Skype, MSN and Line), and no mention was made of WhatsApp or Zoom. The Skype software program was largely used because it is broadly available for all platforms (26) and free of charge (24, 25).

A first technological barrier identified in the analysis was the recurrence of technical problems encountered, such as an audio lag or a call disruption, which affected greatly video calls use. The development and generalization of new technologies and Wi-Fi seem to have solved most of those problems. However, the evolution of digital tools and services has brought new ergonomic issues, which also impact their usability and accessibility. In the selected articles, OAs who participated in video calls had to use computer, tablet or smartphone that required tactile interaction, and thus, more complex interfaces. OAs generally found difficult to use the touch screen, but also found the devices too heavy. However, the authors made no mention of usability issues caused by the software programs interfaces.

A possible solution to enhance the first experience of the video calling technology by OAs could be to identify those ergonomic and technical problems, early in the intervention, by conducting user tests with OAs. These tests would help to make the video calling technology more accessible to older users, either by providing ergonomic adaptations if needed (e.g., sensor pens, tablet support), or by choosing technology more tailored to OAs' needs such as a mobile telepresence robot (47), or a TV (25).

Apart from videophones, most of the OAs seemed to need training in the basic knowledge of video calling technologies. These training programs need to be adapted to OAs' cognitive capacities. Quillion-Dupré (55) created an adapted training program for OAs to use a tablet-based digital agenda. She designed an errorless training with spaced retrieval, a method proven to be more efficient than classical techniques such as trial-and-error learning, especially with OAs with memory impairment (56, 57). Czaja and Sharit (58) provided several recommendations on good practices for designing training that is appropriate for OAs. For a training program to be useful and effective, the form (individual/collective; face-to-face/online; with/without manual; paper/digital manual; formal/informal), the length of the program, the frequency and duration of the training sessions, the location in which the training takes place (home, association…), the pace within the training sessions (set by the instructor/by the learner), etc., should be considered.

However, although training may resolve several usage problems, some OAs may still face usability issues. In this case, additional support by skilled staff members during video calls sessions should be provided to ensure proper use of the video calling technology.

#### Human-related Barriers and Possible Solutions

The implementation of video calls in geriatric institutions requires considering OAs' socialization needs, as they already have their habits with telephone calls or in-person visits. Thus, OAs could be reluctant to use an additional communication technology. Moreover, OAs' physical, cognitive and sensory disorders may increase their fatigability, which in turn could impact their perceived vulnerability, their self-esteem and their self-efficacy toward video calling technology. Combined with OAs' lack of experience and negative attitude toward technology, those factors may affect video calls use.

Families' motivation was identified as an enabler in video calls use. However, when family members are more reluctant about video calls, or have a low tolerance for operating and technical difficulties, the resident/family member dyad tends to have a low potential of video calls use (22). Mickus and Luz (22) suggest to this end, some criteria to determine the dyad's potential of effectively using video calls services (i.e., dyad with high, contingent, low, or no potential).

A possible solution to better take into account OAs' vulnerabilities and disorders could be to embed video calls use into regular activities such as meals, or entertainments. This solution has the advantage of not adding extra fatigue to the day, as well as to ‘dress-up' the video calling technology (38), allowing a more progressive familiarization to the video calls services.

In addition, it could be interesting to add a purpose to those video calls sessions [e.g., asking OAs to teach their language to foreign people using the video calls services (43)]. The feeling of being useful during the video call seemed to be rewarding for these OAs and motivated them to overcome potential difficulties associated with the video calling technology.

Finally, training and regular support by staff members appeared to be crucial for OAs to understand the video calling technology, as well as to reassure them about their own capacity in using it. In order to include families more easily into video calls use, it could be interesting to provide a similar kind of training and technical support to family members in need of assistance.

#### Organizational Barriers and Possible Solutions

As stated in the literature review by Schuster and Hunter (30), professionals actively contributed to the use of ICT in elderly care institutions (30). Seven authors out of 15 studies emphasized the role of assistance or troubleshooting by facility staff.

However, the shortage of personnel, the frequent turnover, and their high workload were major obstacles to the implementation of video calls in geriatric settings. Indeed, video calls implied additional tasks such as scheduling appointments, or providing technical support to OAs or even to family members. This supplementary workload, together with staff members' lack of experience and low self-efficacy could negatively impact their motivation for video calls sessions.

That is why, in order to counter those barriers, it is important to study the capacity of staff members to use video calls considering their current working conditions (30). Providing staff members with training sessions appeared to be critical before video calls implementation. Once facility staff was familiarized with the video calls systems, they could then provide assistance for OAs and family members. Thus, considering the team configuration required for the implementation of video calls in geriatric contexts, it would be more appropriate to speak of a triad (i.e., resident/family member/facility staff) than of a dyad (i.e., resident/family member). It would be indeed the triad that determines the potential of use of video calls in geriatric institutions. This finding echoes a dimension that has been discussed in detail in the literature and promoted in “person-centered” approaches to dementia care. Within these approaches, the underlying idea is that care is provided within “dementia care triads” involving the OA with dementia, the informal carer and the health or social care professional (59).

Finally, actively involving staff members into video calls activities could increase their motivation to use this service. In the study by Zamir et al. (46), video calls were integrated into an inter-residential quiz competition, where professionals were major actors. Thus, these professionals welcomed this intervention and were motivated to participate, a feeling that did not seem to be shared by those who only managed technically the calls between the OAs and their families. It might be interesting to test the benefit of integrating video calls in other occupational or therapeutic activities in a randomized study.

#### Ethical Questions

As stated above, video calls use could be hindered by numerous barriers, which were sometimes difficult to overcome. First, there is the question of providing equal and non-discriminatory access to the service.

In some cases, family members or professionals assumed *a priori* that the OA, because of physical and/or cognitive limitations, would be unable to use the technology, without giving him/her the opportunity to try it out, resulting in the exclusion of some people from the intervention. For instance, in one institution, residents with hearing impairments were not recruited by staff members to take part in the video call activity, and thus, missed the opportunity to try and potentially benefit from the intervention (25). A more inclusive and facilitative attitude was observed in other studies, for instance, in another care home, a non-verbal OA had the opportunity to enjoy video calls using lip-reading and sign language. For future works, in order to provide an equal access to video calling technology, it could be interesting to propose it to residents who want to take the opportunity to try the service, regardless of their limitations and, during the tests, to identify the types of adaptations of the activity required to make it accessible to each individual.

A second ethical theme relates to the respect of privacy and autonomy. Indeed, some OAs expressed concerns about video calls systems, evoking security issues [such as having their identity stolen by hackers (26, 47)], or the lack of control over the technology [less control of their image (47)]. To reassure OAs about the technology, accessible information should be provided on the way that video calls services work regarding the respect of privacy and safety, during training and informational programs offered to OAs and to the other actors.

A third theme concerns the balance between benefits and risks of video calls for vulnerable persons, such as OAs with dementia. From the data analyzed, it is not certain that the concept of video calls was well understood for these users. Indeed, several residents suffering from dementia seemed to remember details of the conversation, the interlocutors, but not the context of the exchanges (46). Cognitive deficits may challenge the ability of these individuals to understand the concept of video calls, i.e., talking to a person who is not physically present. Some OAs with dementia have expressed confusion and anxiety when the video calling technology was introduced into their room (25). In some cases, this misunderstanding and confusion introduced by the technology were avoided by the presence of a traditional telephone handset (25). As this handset was the very symbol of remote communication, its presence allowed OAs to understand the purpose of the technology and thus, to use it with confidence. Moreover, explaining and reminding the purpose of the activity before each session could also help the OA to understand the situation. Teams implementing this kind of intervention should take the necessary measures to ensure OAs' satisfaction and pleasure during those sessions, and to make sure that they do not suffer from side-effects (anxiety, confusion). It could be useful to implement regular staff meetings that enables members to exchange about their experiences of video calls with OAs and family members. Staff members could also discuss the benefits-risks balance for each OA taking part in this activity.

Finally, the risk of substitution of physical visits by video calls was another ethical theme identified in the analysis. This risk was reported by family members (26) as well as OAs (45). Several works have discussed how this kind of technology-based care-related interventions should promote and enhance human contact rather than threaten it (60–62). Regarding this issue, a solution suggested is that professionals who administer video calls activities monitor the balance between the modalities of social contact that are offered to older adults in an institution. Broadly, it is recommended to include these ethical considerations in the implementation and impact assessments of video call technologies in care contexts.

### Contributions of the Study

One of the main contributions of this work was to conduct a multidimensional qualitative analysis of the literature on the use of video calls in geriatric institutions using the framework provided by the EUnetHTA Core model, version 3.0 (35). This methodology allowed us to examine the factors involved in the implementation of video calls with institutionalized OAs from multiple perspectives, and in a comprehensive way.

Moreover, in the selected articles from the literature review, video calls use has encountered several barriers at different steps of its implementation in geriatric institutions. This analysis helped us to suggest concrete recommendations for each stage of the process: the preparation, the conduct of sessions, and the evaluation of video calls use in geriatric settings. These suggestions are presented in the next subsection.

#### Authors' Recommendations for the Implementation of Video Calls Interventions in Geriatric Settings

##### Planning Stage

- Discuss with staff members how video calls interventions could help meet residents' social needs and how could this intervention be integrated into the facility's activity programs.

- Allow staff members involved in the implementation of the intervention an adequate time for planning and information.

- Identify one or two project referents, among the facility staff members, that undertake the coordination of the interventions and can provide the leadership necessary for successful implementation.

- Carefully examine available technological solutions available for video calls and choose the one that seems to best meet the needs of residents, family members, and staff in terms of accessibility, ease of installation and use, costs, training needs, data security and privacy issues, technical assistance needs, and sustainability.

- Identify the residents and families interested in the intervention, or who could potentially benefit from it, and present the project to them in a clear and precise manner (e.g., technology, modalities).

- Define an individual plan of socializing activities for each resident who will benefit from video calls, ensuring a balance between remote and direct social contacts.

- Set up a training program for the residents who will participate in the video call activity that is adapted to their needs and abilities. This may be the opportunity to conduct some usability tests and define the necessary adaptations to ensure the accessibility of the activity (technical or human).

- Offer to professionals and members of the resident's family or friends, interested in the activity, specific training on the use of the device. The availability of pedagogical material adapted to this objective (e.g., tutorial) can help to improve the understanding of the use of the system and its adoption.

- Define a mode of use of video calls that will allow for the privacy of the participants (even if a professional must be present during the call).

- Define with the professionals who will manage the activity a strategy for the handling of technical problems that will allow, on the one hand a quick resolution, and on the other hand to calm and reassure the residents and their family members.

##### Conduct of Sessions

- Solicit family members and members of the resident's entourage interested in using the video calling device early enough to schedule an accurate time for the call.

- Respect the schedule that has been agreed upon for the video call with residents and their family members or friends to avoid frustrations or unrealistic expectations (e.g., wanting to use the service at any time when the professionals coordinating the activity or the device are not available).

- Before initiating a video calls session explain again how to use the device and how the session is going to run.

- Monitor the use of the system during the video call session to make the necessary technical or ergonomic adaptations (e.g., volume level, video viewing).

- Monitor resident's behavior during the video call to identify any signs of confusion or stress and adapt the activity accordingly.

##### Assessment

- Define a way of monitoring the activity at the individual and at the institution scale to identify the necessary modifications, whether at the technical, training or psychosocial impact level. A follow-up activity sheet can be used for this purpose.

- Include the video call activity in the team debriefings and evaluation sessions to allow professionals to discuss, on the one hand, about individual and organizational impacts of the intervention, and to define ways to improve the implementation of the device, on the other hand.

- Keep a regular check on the updates of the technologies and applications allowing the conduct of video calls in order to always have a stable, robust and secure system at disposal.

### Limitations of the Study

In this review, the thematic analysis was based on the EUnetHTA Core Model® (35). Thus, data were coded and interpreted according to EUnetHTA domains, topics and issues, used as a set of pre-defined codes to guide the process of data interrogation and organization. However, the use of this model for thematic coding was not straightforward as the different dimensions of the model intersect and complement each other. A proposition in the text of the original publication included in our analysis, which constituted our primary data, referred in many cases to multiple dimensions or topics of the model. Consequently, the coding was done using all the relevant categories from different dimensions, but the presentation in the results section required the proposition to appear within one or another dimension, whose pertinence was decided by consensus. The model certainly provides a very interesting guide for understanding the use and impact of health technologies, and for analyzing scientific publications in the field, but its application requires an important degree of discussion and consensus among researchers.

Another limitation encountered refers to the selection of publications for the review. First, only publications in English and French were selected. Second, it is also possible that studies that did not mention video calls or elderly care institutions in the key words or in the abstract were not included. Third, the literature review did not take into account the quality of the intervention, or the study described, since we gave priority to include as many relevant publications as possible. Thus, some factors that we know are important for the understanding of the interventions or for the appreciation of their impact were not considered in our analysis (e.g., inclusion of a control group, sample sizes, proper description of health status of participants). These aspects limit the generalizability of our results.

## Conclusion

The isolation and loneliness of OAs in institutions are a problem that has been particularly discussed lately with the successive confinements and restrictions due to the COVID-19 epidemic. Video calls have been one of the solutions proposed by several geriatric institutions to maintain the social link between residents and their families. This literature review has shown that this technology can help connect OAs with their loved ones who are unable to travel. Generally speaking, when the family and the resident perceive the usefulness of video calls, such as having richer exchanges, this service reduces the feeling of loneliness in the OAs and improves the quality of social interactions within the family.

However, the level of acceptance of video calls by the residents, their families and the facility staff varies according to the studies. Various organizational, human-related, ethical and technological barriers and proposed solutions were also identified. Future research must better take into account the family and the facility staff perspectives and needs in the implementation and the study of the acceptance of video calls in institutions. In the future, health economics, organizational, ethical and legal aspects should be better described and addressed. Finally, we highlight the importance of conducting small pilot tests before the implementation of video call services in geriatric institutions that can be helpful to identify technical, human-related, organizational or ethical requirements at the institutional and the individual level.

## Data Availability Statement

The original contributions presented in the study are included in the article/Supplementary Material, further inquiries can be directed to the corresponding author/s.

## Author Contributions

BN, A-SR, and MP: conceptualization and methodology, writing—original draft preparation, and writing—review and editing. BN: formal analysis and investigation and project administration. All authors have read and agreed to the published version of the manuscript.

## Funding

This work was supported by Technosens (Grenoble), CIFRE funding (2021/0344), Assistance Publique-Hôpitaux de Paris (AP-HP) and Université de Paris.

## Conflict of Interest

The authors declare that the research was conducted in the absence of any commercial or financial relationships that could be construed as a potential conflict of interest.

## Publisher's Note

All claims expressed in this article are solely those of the authors and do not necessarily represent those of their affiliated organizations, or those of the publisher, the editors and the reviewers. Any product that may be evaluated in this article, or claim that may be made by its manufacturer, is not guaranteed or endorsed by the publisher.
