# Supplementary material for: Video Calls for Older Adults: A Narrative Review of Experiments Involving Older Adults in Elderly Care Institutions
Source: Front Public Health. 2022 Jan 14;9:751150. doi: 10.3389/fpubh.2021.751150 (PMC8795665; doi:10.3389/fpubh.2021.751150)
Supplement: Supplementary file 1 [file Table_1.pdf]

Supplementary Materials

1 Supplementary Tables

Table 1. A summary of the methodological features of the selected studies

Study	Sample	Study design	Assessment objective	Assessment Indicators (Method)	Benefits of video call use	Impact on maintaining social interactions
Mickus and Luz (2002) (22)	Healthy and MCI (n=10)	Exploratory Qualitative	Feasibility and technology acceptance (Quality and frequency of calls)	Frequency of calls, Perceived usefulness, Ease of use, Satisfaction (Likert scales and open-ended questions)	Satisfaction with videophone use	Enhanced the quality of social interactions, adding a new value (video)
Sävenstedt *et al.* (2003) (45)	Dementia (n=7)	Exploratory Qualitative	Technology acceptance<br><br>(Quality of communication)	Experiences with video-phone (interviews)	- Facilitated and enhanced social interactions<br>- Reduced negative feelings (guilt) (FMs)	OAs more focused and involved during conversations
Hensel *et al.* (2007) (39)	Mobility limitations (n=1)	Case study	Technology utility and usability	Perceived advantages and disadvantages, Experiences of family members in NH placement	- Increased social presence<br>- Enhanced the quality of social interactions<br>- Enjoyment	- Increased social presence (video)<br>- Enhanced affective communication

				(semi-structured interview)	- OAs more involved in the family life	
Demiris *et al.* (2008) (40)	NP but “mentally competent” (n= 4)	Exploratory Qualitative	Technology acceptance (psychosocial impact), usability, utility	Technical quality, Usability (questionnaire), Perceived utility, Type of conversations, Quality and frequency of communication, Stress, Isolation and Loneliness (semi-structured interview)	- Reduced negative feelings (guilt) (FMs)<br>- Enjoyment	- Enhanced social presence and quality of social interactions, connectedness<br>- Helped to reduce feeling of isolation and loneliness
Tsai *et al.* (2010) (27)	Healthy and cognitive impairment (IG n=24; CG n=33)	Quasi Experimental (CG: Receive regular visits)	Clinical impact (psychological impact)	Social support (SSBS), Loneliness (UCLA Loneliness Scale), Depressive status (GDS), Number of calls, number of visits	- Enhanced emotional and appraisal social support<br>- Reduced loneliness<br>- Less depressive symptoms	- Helped to reduce feeling of loneliness
Tsai and Tsai (2011) (23)	Healthy and cognitive impairment (IG n=40; CG n=50)	Randomized longitudinal trial (CG: Receive regular visits)	Clinical impact (psychological impact)	Social support (SSBS), Loneliness (UCLA Loneliness Scale), Depressive status (GDS), Number of calls, number of visits	- Enhanced emotional and appraisal social support<br>- Reduced loneliness<br>- Less depressive symptoms	- Helped to reduce feeling of loneliness<br>- Enhanced social presence

Siniscarco *et al.* (2017) (24)	Healthy and cognitive impairment (n=8)	Exploratory quantitative & qualitative	Clinical impact (impact on loneliness)	Companionship, emotional loneliness, social isolation, opportunities of nurturance, emotional support, informational support, geriatric depression, video conferencing use (scales)	- Happiness<br>- More involved in the family life	NA
Zamir *et al.* (2018) (25)	Healthy, cognitive impairment, non-verbal (no dementia) (n=18)	Collaborative Action Research (CAR)<br><br>Ethnographic	Feasibility and acceptance	Usability, SoW aesthetics, attitudes, care environment, loneliness and social isolation (observation, unstructured interview, form, memo writing, reflective diary)	- OAs more involved in the family life<br>- Enjoyment	NA
Chiu and Wu (2019) (41)	NP but no dementia (CG n=17; Group 1 n=19; Group 2 n=18)	Randomized trial (CG: Receive conventional care)	Clinical impact (social support, psychological well-being, quality of life) and user experience	Health-related quality of life (SF-12), Social support (TISSB), Happiness (CHI), Depression (CES-D), Cognitive function (SPMSQ), Physical	- Better quality of life (better in Group 2)<br>- Better social support<br>- Happiness<br>- Less depressive symptoms	Better social support with family, friends and staff members

				functional status (IADL)		
Moyle *et al* (2019) (47)	Healthy and cognitive impairment (no dementia) (n=6)	Exploratory qualitative	Feasibility	User Experience and usability (post-intervention semi-structured interview)	NA	NA
Niebler *et al.* (2019) (26)	Cognitive impairment (n=41)	Exploratory qualitative	Acceptability	User Experience (Semi-structured interviews)	- Reduced negative feelings (FMs)<br>- Enjoyment<br>- Enhanced patient-relative relationship	NA
Tsai, *et al.* (2020) (42)	Healthy and cognitive impairment (no dementia) (IG n=32; CG n=30)	Randomized trial (CG: Receive regular visits)	Clinical impact	Feelings of loneliness (UCLA Loneliness Scale), Depressive symptoms (GDS), Quality of life (SF-36)	- Reduced loneliness<br>- Better quality of life (pain, vitality, physiological health)	Enhanced quality of social interactions
Sacco, et al. (2020) (44)	Healthy and cognitive impairment (n=132)	Cross-sectional	Utility, usability and acceptance	Ability to establish communication (observation), Preferred virtual communication mode, Satisfaction (Likert scale)	NA	NA

Carcavilla *et al.* (2020) (43)	Healthy<br>(IG n=21; CG n=25)	Randomized trial<br>(CG: Participate in social activities)	Clinical impact	Self-esteem (Likert scale), Positive and negative affects (Scale)	Improves self-esteem	NA
Zamir et al. (2020) (46)	Healthy and cognitive impairment (n=22)	Collaborative Action Research (CAR)<br><br>Ethnographic	Clinical impact, Feasibility and Acceptability	Description of calls, Quality of the intervention (feedback form, observations, semi-structured interview)	- OA with dementia remembered some faces, conversations and the activity<br>- Happiness<br>- Enhanced social interactions<br>- Overcome boredom<br>- Increases social connectedness (among residents, SMs, residences)	- Enhanced quality of social interactions<br>- Increased social connectedness (between residents, SMs, residences)

*CES-D = Center for Epidemiologic Studies Depression scale; CHI = Chinese person's Happiness Inventory; FMs = Family Members; GDS = Geriatric Depression Scale; IADL = Instrumental Activities of Daily Living; MCI = Mild Cognitive Impairment; n = number; NA = Not Applicable; NH = Nursing Home; NP = Not Precised; SF-12 = 12-item short form health survey; SF-36 = 36-item short form health survey; SMs = Staff Members; SoW = Skype on Wheels; SPMSQ = Short Portable Mental Status Questionnaire; SSBS = Social Support Behaviors Scale; TISSB = Taiwanese Inventory of Social Supportive Behavior; UCLA = University of California Los Angeles
