# Supplementary material for: Video Calls for Older Adults: A Narrative Review of Experiments Involving Older Adults in Elderly Care Institutions
Source: Front Public Health. 2022 Jan 14;9:751150. doi: 10.3389/fpubh.2021.751150 (PMC8795665; doi:10.3389/fpubh.2021.751150)
Supplement: Supplementary file 2 [file Table_2.pdf]

Table 2. General description of the technologies used and the setting of the studies

Study	Device	Ergonomic adaptation	Tools required	Role of SMs	Role of researchers	Tool's availability	Setting of use	Frequency of use	Technical issues
Mickus and Luz (2002) (22)	Videophone	✓	Telephone line	Resolve technical issues	Help for the first video call, Resolve technical issues	Unlimited	Resident's room	NP	- Interruption of video connection<br>- No image<br>- Freezing image
Sävenstedt *et al.* (2003) (45)	Videophone	✗	Telephone line or ADSL	Receive the calls, Resolve technical issues	✗	Punctual	Resident's room	Once a day (during 3 to 18 months)	NP
Hensel *et al.* (2007) (39)	Videophone	✗	Telephone line	✗	✗	Unlimited	Resident's room	Once a week (during 3 months)	- Audio delays<br>- Small screen<br>- Usability issue
Demiris *et al.* (2008) (40)	Videophone	✗	Telephone line	NP	NP	Unlimited	Resident's room	Once a week (during 3 months)	- Audio delays
Tsai *et al.* (2010) (27)	Laptop	✗	Wi-Fi MSN or Skype	NP	Help during calls	Punctual	NP	Once a week (during 3 months)	NP

Tsai and Tsai (2011) (23)	Laptop	✗	Wi-Fi MSN or Skype	Help during calls	Help for the first 3 months	Punctual	Resident's room	Once a week (during 12 months)	NP
Siniscarco *et al.* (2017) (24)	Tabletop	✓	Wi-Fi Skype	✗	Teach regularly	Unlimited	Resident's room	Once a week (during 2 months)	- Internet disconnections<br>- Ergonomy of buttons
Zamir *et al.* (2018) (25)	Tablet	✓	Wi-Fi Skype	Assist	✗	Punctual	Resident's room	Once or twice a month (during 15 months)	- Not user-friendly<br>- Poor sound quality<br>- Internet disconnections<br>- Small screen
Chiu and Wu (2019) (41)	Tablet	✓	Wi-Fi Line	✗	Teach	Limited	NP	Once a week (during 12 weeks)	NP
Moyle *et al* (2019) (47)	Tablet	✗	Wi-Fi Skype	✗	Help and train	Punctual	NP	NA	- Heavy<br>- Low volume<br>- Usability
Niebler *et al.* (2019) (26)	Tablet	✓	Wi-Fi Skype	Assist	✗	Punctual	Resident's room	NP	- Difficulties in placing oneself in front of the camera
Tsai, *et al.* (2020) (42)	Smartphone	✗	Wi-Fi Line	Assist	✗	Unlimited	Everywhere	Once a week (during 6 months)	NP
Sacco, et al. (2020) (44)	NP	NA	NA	NA	NA	NA	NA	NA	NA

Carcavilla *et al.* (2020) (43)	TV	✗	Wi-Fi Skype	NP	✗	Punctual	Dedicated room	Twice a week (during 2 weeks)	NP
Zamir et al. (2020) (46)	Tablet (SoW) TV	✓	Wi-Fi Skype	Make the calls	Resolve technical issues	Punctual	Communal area	Once a month (during 8 months)	- Small screen (tablet)<br>- Cumbersome (tablet)<br>- Disconnections (TV)

ADSL = Asymmetric Digital Subscriber Line; MSN = Microsoft Social Network (Windows Live Messenger) ; NA = Not Applicable; NP = Not Precised; SMs = Staff Members; SoW = Skype on Wheels
