# Supplementary material for: Video Calls for Older Adults: A Narrative Review of Experiments Involving Older Adults in Elderly Care Institutions
Source: Front Public Health. 2022 Jan 14;9:751150. doi: 10.3389/fpubh.2021.751150 (PMC8795665; doi:10.3389/fpubh.2021.751150)
Supplement: Supplementary file 3 [file Table_3.pdf]

Table 3. A summary of key barriers, enablers and suggested solutions for future studies/research

Domain	Key barriers to video call use	Enablers to video call use	Solutions for good implementation
Technological	- Technical issues (e.g. audio lag, internet disconnection…)<br><br>- Accessibility and usability of the technology<br><br>- Lack of support for OAs<br><br>- Incapacity of OAs for using the technology alone<br><br>- Lack of adapted training for OAs, FMs and SMs	- Make ergonomic adaptations (tablet support, sensor pen, …)<br><br>- Providing technical support for both OAs and SMs<br><br>- Using modern technology such as telepresence robot, or a bigger screen such as TV<br><br>- Providing a training for OAs, FMs and SMs	- Pre-test the technology with OAs to identify possible ergonomic and technical problems, thus potential ergonomic and technical corrections/adaptations<br><br>- Design and implement an errorless training with spaced retrieval for using the technology for OAs<br><br>- Providing a regular assisted /facilitated use for OAs who will still encounter problems using the technology but who still want to benefit from the activity
Human-related	- Socialization needs already met (in-person visits, telephone calls)<br><br>- Fatigability of OAs (cognitive, physical, sensory disorders)<br><br>- OAs' perceived vulnerability against the new technology<br><br>- Low self-esteem and self-efficacy<br><br>- Lack of experience with the technology	- Embedding video calls use into regular activities to 'dress-up' the technology (meals, entertainment)<br><br>- Giving a goal to video calls (teach a new language) to engage motivation and feeling of reward<br><br>- Providing technical support for OAs and FMs via skilled staff members (via nurses station for example)	- A minimum of one person in the institution needs to be expert, or at least aware of technical issues in order to provide regular support to reassure OAs<br><br>- Design a specific training/support, or an informational session for FMs to familiarize them with video calling technology

	- Negative attitude towards the technology<br>- Lack of FMs commitment on the use of video calls (social influence)<br><br>- The use of the technology is too demanding (cognitive load) and stirs strong negative feelings (emotional load)<br><br>- Dependence of FMs or OAs over SMs		
Organizational	- The organization and delivery of video calls activities is a complex process (taking appointments, preparing materials, providing assistance, troubleshooting)<br><br>- For SMs the activity may result in additional workload<br><br>- Shortage of personnel in institutions<br><br>- Important SMs turnover (loss of key video call information)<br><br>- SMs lack of experience with the technology and low self-efficacy<br><br>- SMs who are not interested in video calls use	- Providing punctual training or informational sessions for SMs<br><br>- Arouse SMs' interest by giving them an active role	- As video calls use depends on SMs' engagement, first embed video calls into daily activities (as part of an entertainment or a competition) would engage SMs in future calls with FMs<br><br>- Study the capacity of SMs to use video calling technology in the current working conditions in order to assess the feasibility of the implementation

Ethical	- Discrimination in the' selection of participations for video calls sessions (considering *a priori* that the person will not be able to use the system before trying)<br><br>- OAs' security concerns and lack of sense of control and confidence in the technology<br><br>- Privacy issues<br><br>- Confusion and agitation of cognitively impaired OAs (difficulty in understanding the concept of video calls)<br><br>- Fear of in-visits replacement by video calls	- Giving all OAs who want to use the system the opportunity to try out (regardless of their limitations)<br><br>- Providing OAs accessible information about the functioning of the system and how data is treated and secured<br><br>- Adding a handset near the video calling technology to enhance the comprehension of the activity/system	- Regular informational sessions to reassure OAs about privacy concerns<br><br>- Explain before each video call the purpose of the technology, as it does not echo OAs' representation of a communication technology (such as the telephone)<br><br>- Examine OAs' satisfaction during the call to avoid side-effects<br><br>- Implement regular meetings in order for SMs to exchange about their experiences with video calls use. Benefit-harm and in-person virtual communication balance could be discussed
FMs = Family Members; OAs = Older Adults; SMs = Staff Members			
